# The prevalence of headache in the adult population of Morocco: a cross-sectional population-based study

**DOI:** 10.1186/s10194-024-01761-y

**Published:** 2024-04-03

**Authors:** Najib Kissani, Latifa Adarmouch, Aboubacar Sidik Sidibe, Abderrahmane Garmane, Rachid Founoun, Mohamed Chraa, Hallie Thomas, Andreas Husøy, Timothy J Steiner

**Affiliations:** 1 Laboratory of Clinical and Experimental Neuroscience, Faculty of Medicine, Cadi Ayyad University, Marrakech, Morocco; 2 Department of Neurology, Mohammed VI University Hospital, Marrakech, Morocco; 3 Community Medicine and Public Health Department, Bioscience and Health Research Laboratory, Faculty of Medicine, Cadi Ayyad University, Marrakech, Morocco; 4 Marrakech Medical School, Marrakech, Morocco; 5 Department of Neuromedicine and Movement Science, Norwegian University of Science and Technology, Edvard Griegs gate, Trondheim, Norway; 6 Department of Neurology, University of Copenhagen, Copenhagen, Denmark; 7 Division of Brain Sciences, Imperial College London, London, UK

**Keywords:** Epidemiology, Population-based study, Prevalence, Headache, Migraine, Tension-type headache, Medication-overuse headache, Morocco, Maghreb, Eastern Mediterranean Region, Global Campaign against Headache

## Abstract

**Background:**

The series of population-based studies conducted by the Global Campaign against Headache has, so far, included Pakistan and Saudi Arabia from the Eastern Mediterranean Region. The Maghreb countries of North Africa, also part of this Region, are geographically apart and culturally very different from these countries. Here we report a study in Morocco.

**Methods:**

We applied the standardised methodology of Global Campaign studies, with cluster-randomized sampling in regions of Morocco selected to be representative of its diversities. In three of these regions, in accordance with this methodology, we made unannounced visits to randomly selected households and, from each, interviewed one randomly selected adult member (aged 18–65 years) using the HARDSHIP structured questionnaire translated into Moroccan Arabic and French. In a fourth region (Fès), because permission for such sampling was not given by the administrative authority, people were randomly stopped in streets and markets and, when willing, interviewed using the same questionnaire. This was a major protocol violation.

**Results:**

We included 3,474 participants, 1,074 (41.7%) from Agadir, 1,079 (41.9%) from Marrakech, 422 (16.4%) from Tétouan and 899 from Fès. In a second protocol violation, interviewers failed to record the non-participating proportion. In the main analysis, excluding Fès, observed 1-year prevalence of any headache was 80.1% among females, 68.2% among males. Observed 1-day prevalence (headache yesterday) was 17.8%. After adjustment for age and gender, migraine prevalence was 30.8% (higher among females [aOR = 1.6]) and TTH prevalence 32.1% (lower among females [aOR = 0.8]). Headache on ≥ 15 days/month (H15+) was very common (10.5%), and in more than half of cases (5.9%) associated with acute medication overuse (on ≥ 15 days/month) and accordingly diagnosed as probable medication-overuse headache (pMOH). Both pMOH (aOR = 2.6) and other H15+ (aOR = 1.9) were more common among females. In the Fès sample, adjusted prevalences were similar, numerically but not significantly higher except for other H15+.

**Conclusions:**

While the 1-year prevalence of headache among adults in Morocco is similar to that of many other countries, migraine on the evidence here is at the upper end of the global range, but not outside it. H15 + and pMOH are very prevalent, contributing to the high one-day prevalence of headache.

## Background

Over the last two decades, knowledge of the global burden of headache has been substantially augmented [1–3]. Nevertheless, areas of the world remain where reliable data are sparse. It is a primary purpose of the Global Campaign against Headache to fill these knowledge gaps [2].

The Maghreb countries of North Africa are one such area. These include the Kingdom of Morocco, the fourth most populous Arab country, with just over 37 million people [4]. The Maghreb is part of the Eastern Mediterranean Region, in the terminology used by the World Health Organization (WHO). Within this Region, the Global Campaign has conducted population-based studies in Pakistan [5] and Saudi Arabia [6]. However, Morocco is geographically far apart and, due to population dominance of Amazigh over Arab, ethnically and culturally very different from these countries.

Morocco has both Atlantic and Mediterranean coastlines, along with large mountainous regions, while most of the southeast of the country is in the Sahara Desert. Its people are a cultural mix of Amazigh, Arab, Jewish, African and European [7]. Agriculture provides about 40% of its employment, but it has high unemployment, especially among youths [4]. It is classified by the World Bank as a lower-middle-income country [8]. There are no published data from Morocco regarding headache prevalence. Evidence from other Arab countries, in the main far wealthier (such as Saudi Arabia, Kuwait and Qatar), is that migraine is as prevalent in these as in western countries [9]. Our own data from Saudi Arabia show both migraine and tension-type headache (TTH) to be more prevalent than their global averages [6]. These data cannot be extrapolated to Morocco.

The aim of this study, therefore, was to estimate the prevalences and demographic associations in Morocco of migraine and TTH, and of the group of headache disorders characterized by headache on ≥ 15 days/month (H15+), which include medication-overuse headache (MOH). It was conducted as one of the Global Campaign’s series of population-based studies using standardized methodology [10] and questionnaire [11].

## Methods

### Ethics and approvals

The protocol and questionnaire were approved by the Comité d’Ethique pour la Recherche Biomédicale of Centre Hospitalier Universitaire Ibn Rochd Casablanca, Morocco.

The study was conducted in accordance with the Declaration of Helsinki [12], and with Moroccan regulation concerning the exercise of medicine.

Administrative authorizations were required for house-to-house surveys, and were obtained from the regional administrative authorities of Agadir, Marrakech and Tétouan.

All participants gave verbal consent to inclusion. Data were gathered anonymously, and managed in accordance with European and Moroccan data protection legislation.

### Study design

This was a cross-sectional study of the adult general population of the country. The standardized methodology has been published in full [10], and is summarized here, along with details specific to Morocco. Trained interviewers (medical graduates or senior medical students) used a structured questionnaire in face-to-face interviews during February to September 2019.

#### Sampling

The study aimed for a random sample through four-level cluster sampling: region, district, household and individual.

Four of the country’s geographical regions were selected having regard to the country’s geographic, ethnic and cultural diversities, but also with regard to population density, feasibility of access and interviewers’ personal safety. These four regions were Tétouan in the coastal lowlands of the north, Fès in northern inland Morocco, Marrakech in central Morocco, west of the foothills of the Atlas Mountains, and Agadir in the south.

Regions were sampled according to their population size and urban/rural divide, as evidenced in January 2019 by the Recensement Général de la Population et de l’Habitat 2004 [13]. Districts were randomly selected from published lists available for each region. Sampling commenced with the first dwelling to the right of the central administrative building of each district and continued in a line, with unannounced visits, until 30 eligible individuals had been identified. If the door of a dwelling did not open, interviewers proceeded to the next in line. Within each dwelling, one member aged 18–65 years was randomly selected from each household, defined as a group of individuals living together and sharing a kitchen. In dwellings with more than one biologically unrelated household, each was sampled.

A total sample of *N* = 3,600 was intended, well exceeding the minimum recommended in guidelines [10]. This required sampling in a total of 120 districts.

In Fès, administrative authorization was not provided (there was no response to the request), which precluded house-to-house visiting. The interviewers interrogated willing people in the streets, cafes and marketplaces of selected districts. This was an invalidating violation of protocol; data from Fès were therefore analysed separately.

#### Data collection

Each region was allocated a group of 3–4 interviewers (total 12), either medical graduates or senior medical students, led by a study coordinator.

Interviewers used modules from the Headache-Attributed Restriction, Disability, Social Handicap and Impaired Participation (HARDSHIP) questionnaire [11], translated into Moroccan Arabic and French (the former used mostly). Demographic enquiry was followed by neutral screening questions (“Have you ever had a headache in your lifetime?” and “Have you had headache during the last 12 months?”). Those responding “yes” to both continued with headache diagnostic and characterization questions, the former based on ICHD-3 [14]. To maintain clarity in this enquiry, participants identifying more than one headache type were instructed to focus on the one they considered most bothersome. Separate questions asked about headache on the preceding day (“headache yesterday” [HY]).

#### Quality control

Interviewers received face-to-face and online instruction about the study, its purpose, and its procedures, along with a survey guide to help them organize their work. Data collection was monitored by the principal investigator and two members of the study team, who assisted interviewers and coordinators by email and phone. After data collection, a random sample of 30 questionnaires from each region (total 120) was checked for accuracy.

### Data entry and verification

All questionnaires were retained and managed in a locked facility in the coordinating centre in Marrakech Medical School, with access limited to the study team.

Two trained research assistants separately entered the data into SPSS spreadsheets, which were compared, with aberrant values corrected by referring to the original questionnaires. Finally, a random sample of 5% of all questionnaires were checked fully against entered data, with very few inconsistencies found.

### Analysis

The primary analysis was of the data from Tétouan, Marrakech and Agadir.

Demographic and social status variables were as follows: gender (either male or female); age (as a continuous variable, and later categorized as 18–25, 26–35, 36–45, 46–55 or 56–65 years); habitation (either urban or rural); marital status (single, married, or divorced or widowed); level of education (none, primary school, secondary school or college/university). These were analyzed descriptively using means ± standard deviations (SDs) or medians as appropriate. Sample distributions of age, gender and habitation were compared to those of the general population in Morocco using one-sample t test and chi-squared tests.

Headache diagnoses were made algorithmically during analysis. Participants with headache on ≥ 15 days/month (H15+) were first identified, and classified either as probable MOH (pMOH) if reporting acute medication usage on ≥ 15 days/month or, if not, as “other H15+”. Remaining participants were classified, in hierarchical order and according to the characteristics of their most bothersome headache and the criteria of ICHD-3 [14], as definite migraine, definite TTH, probable migraine or probable TTH. Only one diagnosis was made per individual.

One-year prevalences of all headache and of each headache type (with definite and probable migraine combined, and definite and probable TTH) were reported as percentages (%) with 95% confidence intervals (CIs). Observed values were then adjusted for age and gender. Point (1-day) prevalence of any headache was calculated from reported HY, and also estimated, as predicted point prevalence, from 1-year prevalence and reported headache frequency in days/month.

Demographic and social status variables were considered as independent variables in association analyses, with headache type as dependent variable. Unadjusted odds ratios (ORs) were calculated in bivariate analysis, and adjusted ORs (aORs) in multivariate analysis, each with 95% CIs.

Significance was set at *p* < 0.05. We used Microsoft Excel to calculate the age- and gender-adjusted prevalences and SPSS version 28 for all other analyses.

Data from Fès were analysed separately, with prevalence estimates, but not included in association analyses.

## Results

A total of 3,474 participants were included, with 1,074 (41.7%) from Agadir, 1,079 (41.9%) from Marrakech, 422 (16.4%) from Tétouan and 899 from Fès. In a second protocol violation, interviewers failed to record refusals; retrospectively, they estimated the non-participating proportion as up to 10%.

In the primary analysis (*N* = 2,575), excluding Fès, 1,038 (40.3%) were male and 1,535 (59.6%) female, with information on gender missing for two participants. Females make up 49.7% of the population of Morocco aged 18–65 years [13], and were therefore over-represented in the sample (chi-squared [1, *N* = 2,573] = 101.9, *p* < 0.001). Mean ages were 40.0 years among males, 39.1 years among females, and 39.5 (± SD = 13.3) years overall, well matching that of Moroccans aged 18–65 years (38.7 years) despite being statistically different (*p* = 0.004). Urbanization in the sample (66.3%) was not significantly different from that of the country (64.0% [13]; chi-squared [1, *N* = 2,529] = 3.0, *p* = 0.08).

**Table 1:** Observed 1-year prevalences by headache type and gender in the main sample (excluding Fès)

Headache type	Overall	Male	Female
	% [95% confidence interval]		
All headache	75.3 [73.6–76.9]	68.2 [65.3–71.0]	80.1 [78.0-82.1]
Migraine definite probable	31.4 [30.0-33.2] 18.5 [17.1–20.1] 12.9 [11.6–14.2]	25.3 [22.7–28.1] 12.5 [10.6–14.7] 12.8 [10.8–15.0]	35.5 [33.1–38.0] 22.6 [20.6–24.8] 12.9 [11.3–14.7]
TTH definite probable	32.1 [30.3–34.0] 28.6 [26.8–30.4] 3.5 [2.9–4.3]	36.2 [33.3–39.2] 32.9 [30.0-35.8] 3.4 [2.4–4.7]	29.3 [27.1–31.7] 25.7 [23.5–27.9] 3.6 [2.8–4.7]
pMOH	6.1 [5.2–7.1]	3.1 [2.1–4.3]	8.1 [6.8–9.6]
Other H15+	5.2 [4.3–6.1]	3.1 [2.1–4.3]	6.6 [5.4–7.9]

TTH: tension-type headache; pMOH: probable medication-overuse headache; H15+: headache on ≥ 15 days/month

### Headache

The 1-year prevalence of all headache was 75.3% (95% CI: 73.8–77.2), higher in females (80.4 [78.3-82-4]) than in males (68.3% [65.4–71.1]). Prevalence of each headache type in the main sample, excluding Fès, is shown in Table 1. Migraine (31.3%) and TTH (32.2%) were similarly prevalent. H15 + was reported by 11.3% of the sample, 6.1% classified as pMOH and 5.2% as other H15+. Unclassified headaches were 0.8%.

Migraine was more common among females (35.4%; OR = 1.6; *p* < 0.001) than males (25.3%). The opposite was true for TTH, which was less prevalent among females (29.4% vs. 36.2%; OR = 0.7; *p* < 0.001). Females were more likely to have pMOH (8.1%; OR = 2.8; *p* < 0.001) or other H15+ (6.6%; OR = 2.2; *p* < 0.001) than males (each 3.1%).

Age- and gender-adjusted prevalence estimates were 30.8% (95% CI: 29.0-32.6) for migraine, 32.1% (30.3–34.0) for TTH, 5.9% (5.0-6.9) for pMOH and 4.6% (3.8–6.9) for other H15+.

HY was reported by 23.6% of those with any headache, yielding a point prevalence estimate (23.6*75.3%) of 17.8%. The predicted point prevalence, from 1-year prevalence and frequency in days/month, was somewhat lower at 14.1%. Proportions reporting HY by diagnosed headache type were 20.3% for migraine, 13.4% for TTH, 53.5% for pMOH and 49.6% for other H15+.

#### Estimates from the Fès sample

In the Fès sample (*N* = 899), males were heavily overrepresented (*n* = 584 [65.0%]; females: *n* = 313 [35.0%]; missing 2). Table 2 shows observed 1-year prevalences of headache and each type by gender, with 3.6% unclassified.

**Table 2:** Observed 1-year prevalences by headache type and gender in the Fès sample

Headache type	Overall	Male	Female
	% [95% confidence interval]		
All headache	81.8 [79.0-84.2]	78.8 [75.2–82.0]	87.9 [83.7–91.3]
Migraine definite probable	30.9 [27.9–34.1] 13.0 [10.9–15.4] 17.9 [15.5–20.6]	27.2 [23.7–31.0] 9.2 [7.0-11.9] 18.0 [15.0-21.3]	38.0 [32.7–43.7] 20.1 [15.8–25.0] 18.0 [14.0-22.7]
TTH definite probable	39.6 [36.4–42.9] 26.0 [23.2–29.1] 13.6 [11.4–16.0]	42.3 [38.3–46.4] 27.2 [23.7–31.0] 15.1 [12.3–18.2]	34.8 [29.6–40.4] 24.0 [19.4–29.2] 10.9 [7.6–14.9]
pMOH	5.8 [4.4–7.5]	3.9 [2.5–5.9]	9.3 [6.3–13.0]
Other H15+	1.9 [1.1-3.0]	1.4 [0.6–2.7]	2.9 [1.3–5.4]

TTH: tension-type headache; pMOH: probable medication-overuse headache; H15+: headache on ≥ 15 days/month

The gender-adjusted estimates are shown in Table 3, alongside those from the main analysis for comparison. The findings in the two samples were not highly discrepant. TTH was the most common headache type in both, although, as already noted, not by a large margin. Nonetheless, except for other H15+ (for which numbers were low), all estimates were higher in the Fès sample than in the main sample, but with the gender-related differentials preserved (Table 3).

**Table 3:** Age- and gender-adjusted 1-year prevalence estimates by headache type in the Fès and main samples

Headache type	Fès sample	Main sample
	% [95% confidence interval]	
Migraine	33.8% [30.7–37.0]	30.8% [29.0-32.6]
TTH	37.0% [33.9–40.3]	32.1% [30.3–34.0]
pMOH	7.5% [5.8–9.4]	5.9% [5.0-6.9]
Other H15+	3.0% [2.0-4.3]	4.6% [3.8–6.9]

TTH: tension-type headache; pMOH: probable medication-overuse headache; H15+: headache on ≥ 15 days/month

**Table 4:** Unadjusted analyses of associations (bivariate analysis) between headache types and demographic and social status variables

Variable	Migraine	TTH	pMOH	Other H15+
	Odds ratio [95% CI]			
Gender				
male (*n* = 1,038)	reference	reference	reference	Reference
female (*n* = 1,535)	1.6 [1.4–1.9] *p* < 0.001	0.7 [0.6–0.9] *p* < 0.001	2.8 [1.9–4.1] *p* < 0.001	2.2 [1.5–3.4] *p* < 0.001
Age (years)				
18–25 (*n* = 473)	reference	reference	reference	reference
26–35 (*n* = 626)	1.1 [0.8–1.4] *p* = 0.59	1.2 [0.9–1.5] *p* = 0.27	1.6 [0.9-3.0] *p* = 0.14	0.9 [0.5–1.5] *p* = 0.60
36–45 (*n* = 604)	1.2 [0.9–1.5] *p* = 0.19	1.1 [0.9–1.5] *p* = 0.33	1.9 [1.0-3.5] *p* = 0.04	0.7 [0.4–1.3] *p* = 0.24
46–55 (*n* = 474)	1.1 [0.9–1.5] *p* = 0.34	1.2 [0.9–1.5] *p* = 0.37	2.6 [1.5–4.9] *p* = 0.001	0.9 [0.5–1.6] *p* = 0.77
56–65 (*n* = 383)	0.9 [0.7–1.2] *p* = 0.49	1.4 [1.0-1.8] *p* = 0.03	2.4 [1.3–4.6] *p* = 0.005	1.4 [0.8–2.4] *p* = 0.28
Habitation				
urban (*n* = 1661)	reference	reference	reference	reference
rural (*n* = 868)	0.9 [0.8–1.1] *p* = 0.60	1.1 [0.9–1.3] *p* = 0.57	0.7 [0.5-1.0] *p* = 0.09	1.0 [0.7–1.4] *p* = 0.99
Marital status				
single (*n* = 626)	reference	reference	reference	reference
married (*n* = 1,752)	1.1 [0.9–1.4] *p* = 0.26	1.0 [0.8–1.2] *p* = 0.63	1.3 [0.9–2.1] *p* = 0.19	1.3 [0.8–2.1] *p* = 0.25
divorced or widowed (*n* = 191)	1.2 [0.9–1.7] *p* = 0.22	0.9 [0.6–1.2] *p* = 0.41	3.1 [1.7–5.4] *p* < 0.001	2.5 [1.3–4.7] *p* = 0.004
Education level				
none (*n* = 1,078)	1.1 [0.8–1.4] *p* = 0.51	0.9 [0.7–1.2] *p* = 0.68	1.5 [0.9–2.6] *p* = 0.10	3.6 [1.7–8.6] *p* = 0.002
primary (*n* = 653)	0.8 [0.6–1.1] *p* = 0.16	1.0 [0.8–1.4] *p* = 0.78	1.1 [0.6–1.9] *p* = 0.82	3.6 [1.7–8.9] *p* = 0.002
secondary (*n* = 436)	0.8 [0.6-1.0] *p* = 0.08	1.3 [1.0-1.7] *p* = 0.11	0.9 [0.4–1.7) *p* = 0.65	2.0 [0.8–5.3] *p* = 0.13
college/university (*n* = 377)	reference	reference	reference	reference

Totals of n for each variable vary because of missing values. pMOH: probable medication-overuse headache; H15+: other headache on ≥ 15 days/month

### Associations

Unadjusted and adjusted analyses of the relationships between headache type and demographic and social status variables are shown in Tables 4 and 5. These confirmed the associations with gender.

Migraine was neither positively nor negatively associated with any of the other variables in either analysis. TTH was most common among the oldest participants (aOR = 1.7; *p* = 0.006) (Table 5). pMOH showed a positive association with increasing age, being most prevalent in those aged 46–55 years (aOR = 2.9; *p* = 0.004) and only slightly less prevalent in those aged 56–65 years (aOR = 2.7; *p* = 0.01) (Table 5). Both pMOH (OR = 3.1; *p* < 0.001) and other H15+ (OR = 2.5; *p* = 0.004) were associated with being widowed or divorced in unadjusted analyses (Table 4) but these lost significance in adjusted analyses (Table 5). Educational level was negatively associated with other H15 + in both analyses (Tables 4 and 5).

**Table 5:** Adjusted analyses of associations (multivariate analysis) between headache types and demographic and social status variables

Variable	Migraine	TTH	pMOH	Other H15+
	Adjusted* odds ratio [95% CI]			
Gender				
male	reference	reference	reference	Reference
female	1.6 [1.3–1.9] *p* < 0.001	0.8 [0.6–0.9] *p* = 0.01	2.6 [1.7-4.0] *p* < 0.001	1.9 [1.2-3.0] *p* = 0.004
Age (years)				
18–25	reference	reference	reference	reference
26–35	1.0 [0.8–1.4] *p* = 0.80	1.3 [1.0-1.7] *p* = 0.10	1.8 [0.9–3.6] *p* = 0.10	0.7 [0.4–1.2] *p* = 0.20
36–45	1.1 [0.8–1.6] *p* = 0.40	1.3 [1.0-1.8] *p* = 0.08	1.9 [1.0–4.0] *p* = 0.07	0.5 [0.3-1.0] *p* = 0.04
46–55	1.1 [0.8–1.5] *p* = 0.65	1.3 [1.0-1.9] *p* = 0.09	2.9 [1.4–6.2] *p* = 0.004	0.6 [0.3–1.2] *p* = 0.15
56–65	0.9 [0.6–1.2] *p* = 0.40	1.7 [1.2–2.4] *p* = 0.006	2.7 [1.2–5.9] *p* = 0.01	0.8 [0.4–1.7] *p* = 0.62
Habitation				
urban	reference	reference	reference	reference
rural	1.1 [0.9–1.3] *p* = 0.60	1.0 [0.8–1.2] *p* = 0.97	1.0 [0.7–1.4] *p* = 0.89	1.2 [0.8–1.8] *p* = 0.32
Marital status				
single	reference	reference	reference	reference
married	1.0 [0.8–1.3] *p* = 0.88	0.9 [0.7–1.1] *p* = 0.28	0.9 [0.5–1.6] *p* = 0.72	1.2 [0.7–2.2] *p* = 0.51
divorced or widowed	1.1 [0.7–1.6] *p* = 0.80	0.8 [0.5–1.2] *p* = 0.22	1.4 [0.7–2.9] *p* = 0.31	1.7 [0.8–3.9] *p* = 0.17
Education level				
none	1.0 [0.8–1.3] *p* = 0.99	0.9 [0.7–1.3] *p* = 0.69	0.8 [0.5–1.5] *p* = 0.55	3.4 [1.5–9.2] *p* = 0.007
primary	0.8 [0.6-1.0] *p* = 0.09	1.0 [0.8–1.4] *p* = 0.81	0.9 [0.5–1.7] *p* = 0.72	3.9 [1.7–10.4] *p* = 0.003
secondary	0.8 [0.6-1.0] *p* = 0.08	1.3 [0.9–1.7] *p* = 0.11	0.7 [0.4–1.5] *p* = 0.38	2.3 [0.9–6.5] *p* = 0.09
college/university	reference	reference	reference	reference

*Adjusted for all other variables. pMOH: probable medication-overuse headache; H15+: other headache on ≥ 15 days/month

## Discussion

This first population-based study of headache in Morocco found very high prevalences among adults aged 18–65 years (essentially, the working population). The observed 1-year prevalence of all headache was 80.4% among females and 68.3% among males. After adjustment for age and gender, migraine prevalence was 30.8%, and positively associated with being female (aOR = 1.6). TTH, by a small margin the most common headache type in our sample, with an age- and gender-adjusted prevalence of 32.1%, was negatively associated with female gender (aOR = 0.8). H15 + was very common (10.5%), and in more than half of cases (5.9%) associated with acute medication overuse (on ≥ 15 days/month) and accordingly diagnosed as pMOH. Both pMOH (aOR = 2.6) and other H15+ (aOR = 1.9) were associated with female gender.

As expected, approximately half of those with H15 + reported HY. The estimated point (1-day) prevalence of headache, based on HY, was 17.8%, or more than one in six of the population, reflecting the high overall prevalence of headache but principally driven by H15+. We believe reported HY, free from recall error, provided a better estimation of the true point prevalence than the (lower) prediction of 14.1% based on (perhaps erroneously) recalled frequency.

In the association analyses, only gender was associated with migraine, in the expected direction (female more than male). TTH was associated with gender in the opposite direction – more common among males than females. Other studies have found this, but it should be noted that participants with migraine, pMOH or other H15+, all more common in females, were included in the control group in the TTH analyses. (For each headache type, cases were compared with non-cases, the latter including all other headache types as well as no headache.)

The Fès sample was two thirds male, which was not unexpected given the method of sampling and the conservative culture of Fès city and region. Statistical correction for this adjusted the prevalence estimate for TTH downwards and all others upwards. With these adjustments, the findings in the Fès sample were not greatly different from those of the main sample. A somewhat higher prevalence of headache overall (81.8% vs. 75.3%) was largely explained by the higher prevalence of TTH in both genders in the Fès sample, although all headache types except other H15+ (with small numbers) were reportedly more common in this sample. This was evidence, in all probability, of interest bias: the principal reason why this method of sampling and engagement is suboptimal is that those with headache are more willingly engaged, and those with troublesome headache even more so.

Morocco is a lower-middle-income country, but, despite real improvements in the last two decades, it has high levels of unemployment and illiteracy [4]. Whether such stressful factors influence headache *prevalence* is uncertain, although poor access to health care does appear to encourage development of H15+ [15]. The 1-year prevalence of all headache (75.3%) was comparable to estimates in many of our previous studies [6, 16–19]; the *proportions* with H15+ (9.0% of males and 18.3% of females) were therefore extremely high. The high prevalence of pMOH, accounting for more than half of H15+, indicated easy access to acute medication despite limited financial means. Unfortunately, we could not explore associations with household income, since we did not expect that participants (among a population tending to be suspicious of such enquiry) would be willing to provide the data.

The very high prevalence of migraine (30.8% when adjusted for age and gender) requires further comment. It is considerably higher than the estimated global prevalence of migraine of 14–15% [1, 20], although the heterogenous study methodologies and diagnostic criteria contributing to global estimates, with a tendency to lower them, have already been the subject of comment [1]. In particular, many published studies clearly excluded probable migraine, while others may have done so without explicitly stating so. Global Campaign studies, using the same methodology as here in the main sample [10, 11], and including probable migraine, have produced estimates from around the world in the range 17.7–34.7% [20], with China being exceptional (9.3% [21]). Morocco is not an outlier from this range, but the 34.7% in Nepal reflected a strong positive association with altitude [16]. This is not the explanation in Morocco, where the high-altitude mountainous regions are thinly populated.

We questioned our finding by looking at responses to the diagnostic question set driving a diagnosis of migraine (Table 6). Notable among those with any headache were the high proportions (in excess of the proportion with migraine) reporting throbbing headache, aggravation by activity and phonophobia, all considered rather specific to migraine. But none stood out as a false driver. Rather, with similar estimates in the two samples, despite very different sampling procedures, it appears that migraine prevalence in Morocco is at the upper end of – but not outside – the global range.

A further possibility is interest bias [12]. Those with headache – and in particular those with more severe headache such as migraine – might have been selectively willing to participate. It is regrettable that the interviewers neglected to record refusals: their later recall of “up to 10%” is inexact and anyway cannot be relied on. Interest bias is more likely if the non-participating proportion was large (> 10%). However, the observed 1-year prevalence of all headache (75.3%) was not unusually high. Further, in the Fès sample, among whom interest bias was more likely, it was TTH prevalence rather than migraine that increased most.

**Table 6:** Responses (among main sample) to the diagnostic question set driving a diagnosis of migraine

Question	Any headache in last year	Classified as definite migraine
	Responding positively (%)	
Duration 4–72 h	71.5	100
Moderate-severe pain	87.8	98.3
Throbbing headache	60.8	65.7
Unilateral headache	36.6	44.6
Aggravation by activity	61.7	85.1
Nausea	22.7	53.3
Vomiting	17.8	40.4
Photophobia	37.7	80.3
Phonophobia	48.4	83.1

### Strengths and limitations

Much care and effort were invested in planning and conducting this study, which nonetheless encountered difficulties. Foremost among these was the slow bureaucratic process of obtaining administrative authorizations from local authorities, necessary in Morocco for door-to-door surveys but never forthcoming in Fès. Even with Fès excluded from the main analysis, the sample size *N* = 2,575 remained adequate [10], although national representativeness was less well assured. The sample had a high female-to-male ratio, for which statistical corrections were made, but reasonably matched the general population of Morocco in terms of age and habitation.

The failure to record non-participants, a serious limitation, has been discussed.

As is the case for all cross-sectional studies with enquiry limited to a single encounter, H15 + could not be further diagnosed beyond recognizing its association, or not, with acute medication overuse [10, 11]. The focus on most bothersome headache when more than one type was reported was necessary to maintain clarity of description [10]. When both migraine and TTH were present, migraine was always likely to be the more bothersome, and be preferentially diagnosed, with resultant underestimation of TTH prevalence. The diagnostic question set was not validated within this study, but had been validated in six earlier studies [22–27] and used previously in 19 languages [11] including both French [28] and Arabic [6].

## Conclusions

The 1-year prevalence of headache in the adult population of Morocco (75.3%) is similar to that of many other countries. However, migraine (30.8%) is, on the evidence here, at the upper end of – but not outside – the global range. Also very high is the prevalence of headache on ≥ 15 days/month (10.5%), over half (5.9%) associated with overconsumption of acute medication. This is a major contributor to the observed point (1-day) prevalence of 17.8%.

This study provides the only population-based data so far available on headache prevalence in Morocco – and in the entire Maghreb region of North Africa. The estimates are therefore the best that can currently be made not only for Morocco but also for the two other ethnically and culturally similar Maghreb countries, Tunisia and Algeria.

## Data Availability

The original data are held on file at Marrakech Medical School, Marrakech, Morocco, and the analytical dataset at Norwegian University of Science and Technology, Trondheim, Norway. Once analysis and publications are completed, they will be freely available for non-commercial purposes to any person requesting access in accordance with the general policy of the Global Campaign against Headache.

## Abbreviations

aOR: Adjusted odds ratio
CI: Confidence interval
d/m: Days/month
GBD: Global Burden of Disease
HARDSHIP: Headache-Attributed Restriction, Disability, Social Handicap and Impaired Participation questionnaire
HY: Headache yesterday
ICHD: International Classification of Headache Disorders
LTB: *Lifting The Burden*
MOH: Medication-overuse headache
OR: Odds ratio
pMOH: Probable MOH
SD: Standard deviation
TTH: Tension-type headache
WHO: World Health Organization
